# Human umbilical cord multipotent mesenchymal stromal cells alleviate acute ischemia-reperfusion injury of spermatogenic cells via reducing inflammatory response and oxidative stress

**DOI:** 10.1186/s13287-020-01813-5

**Published:** 2020-07-17

**Authors:** Liang Zhong, Mengbo Yang, Xiangyu Zou, Tao Du, Huiming Xu, Jie Sun

**Affiliations:** 1 Department of Urology, Shanghai Children’s Medical Center, Shanghai Jiao Tong University School of Medicine, Shanghai, 200127 China; 2 State Key Laboratory of Oncogenes and Related Genes, Renji-Med X Clinical Stem Cell Research Center, Ren Ji Hospital, Shanghai Jiao Tong University School of Medicine, Shanghai, 200127 China; 3 Department of Urology, Henan Provincial People’s Hospital, Zhengzhou City, 450003 China

**Keywords:** Stem cell, Ischemia-reperfusion injury, Spermatogenic cells, Inflammatory response, Oxidative stress

## Abstract

**Background:**

This study was designed to determine the effect of human umbilical cord multipotent mesenchymal stromal cells (hUC-MSC) on acute ischemia/reperfusion (I/R) injury of spermatogenic cells.

**Method:**

The testicular I/R rat model was established through 720° torsion for 1 h. hUC-MSC were intravenously injected 10 min before detorsion. Injury severity of spermatogenic cells was estimated by Johnsen’s score. The proliferating of recipient spermatogonia was measured by the immunostaining of antibodies against Ki67, and all germ cells were detected with DDX4 antibody. And recipient spermatogenesis was assessed by staining spermatozoa with lectin PNA. The levels of inflammatory factors were measured by real-time PCR. And the Selectin-E expression, neutrophil infiltration in the testes was detected by immunostaining. Germ cells apoptosis was tested by TUNEL assay and western blot. Furthermore, the oxidative stress was tested by reactive oxidative species (ROS) levels. In vitro, the condition medium (CM) of hUC-MSC was used to culture human umbilical vein endothelial cells (HUVECs), so as to assess the paracrine effect of hUC-MSC on HUVECs. The protein chip was used to measure the relative concentration of the secretory proteins in the CM of hUC-MSC.

**Result:**

hUC-MSC greatly alleviated the testicular injury induced by testis I/R. The levels of proinflammatory factors were downregulated by hUC-MSC in vivo and in vitro. Neutrophil infiltration, ROS, and germ cell apoptosis in testicular tissues were greatly reduced in the group of hUC-MSC. Paracrine factors secreted by hUC-MSC including growth factors, cytokines, and anti-inflammatory cytokine were rich.

**Conclusion:**

This study demonstrated that intravenously injected hUC-MSC could protect the spermatogenic cells against I/R injury by reducing the inflammatory response, apoptosis, and acute oxidative injury. Paracrine mechanism of hUC-MSC may contribute to the protection of spermatogenic cells against I/R injury. Therefore, the present study provides a method for clinical treatment of attenuate I/R injury of spermatogenic cells.

## Background

Testicular torsion is a common type of urological emergency in adolescent males which usually leads to severe acute ischemia injury of the testis. Previous studies suggest that testicular torsion leads to male infertility because of testicular atrophy [1–3]. Testicular torsion in patients varies in time and degree, and it is commonly believed that the testicular salvage rate is 90% if spermatic cord detorsion is performed within 6 h from the onset of torsion [4]. But detorsion operation itself leads to ischemia/reperfusion (I/R) injury when the blood flow returns to normal. Many cellular and molecular mechanisms are investigated in testicular I/R injury. Inflammatory cascades are the most important pathological mechanism in I/R injury [5]. Apoptotic pathways are activated by inflammation which leads to germ-cell-specific apoptosis [6]. In addition, the production of ROS during I/R also causes DNA damage, endothelial damage, and germinal cell apoptosis [7]. Numerous pharmacological agents have been used as supportive therapy to prevent the adverse effects of I/R injury, including antioxidants, hormones, vitamins, and plant extracts [8]. But most of the agents only targeted to eliminate ROS which is less efficient.

MSCs have been reported to exert therapeutic effects by immunoregulation and suppression of oxidative stress in several diseases [9]. A study reported that MSCs transplanted by local injection into I/R injured testis played a protective effect on germ cells [10]. However, local injection of MSCs may aggravate the intracompartmental pressure of testis, resulting in an occlusion of the microvascular bed that feeds the testicular lobules [11, 12]. On the other hand, intravenous injection might be an appropriate transplantation method for MSCs, which can avoid these risks. Our previous study showed that in I/R injury of the kidney model, intravenous mesenchymal stem cells were mainly blocked in the lung and still exhibited a great protective effect on the kidney [13, 14]. In this study, we explore whether transvenous hUC-MSC attenuate acute I/R injury of spermatogenic cells.

## Methods

### Isolation and culture of hUC-MSC

hUC-MSCs were prepared and identified as described in our previous study [14]. In brief, the umbilical cord was obtained from women undergoing cesarean sections. Women gave informed consent for the collection of their umbilical cord. The collection and subsequent use of the umbilical cord were approved by the Institutional Ethical Review Committee of Shanghai Children’s Medical Center, Shanghai Jiao Tong University School of Medicine. Umbilical cord tissues were cut and then attached to culture plates individually, followed by the addition of minimum essential medium α (MEMα) containing 10% fetal bovine serum (FBS) (All from Life Technology). Approximately 12 days later, the colonies appeared and were cultured on new plastic plates for further expansion. The cells at the third to fifth passage were used in the following experiments.

### Testicular I/R rat model

All works involving animals were approved by Institutional Ethical Review Committee of Shanghai Children’s Medical Center, Shanghai Jiao Tong University School of Medicine. Adult male Sprague–Dawley rats weighing 180 g to 200 g were used. The testicular I/R rat model was established as described [15–17]. As shown in the Fig. S1, testicular I/R rat model was established through rotated 720° in a clockwise direction fixing it to the scrotum with silk suture and maintained for 1 h. Detorsion was performed by untwisting the testis. The success rate of the animal model was up to 80%. Only the testes which return to red from purple or black after detorsion will be used in the further study. 10^7^ hUC-MSC in 0.5 ml PBS were injected into the tail vein 10 min before detorsion (*N* = 5), whereas control animals (*N* = 5) received 0.5 ml PBS instead of the cells. Normal animals were used as the untreated group. The testes tissues were harvested at day 1, day 3, day 7, and day 15 after intervention, respectively.

### Histopathology and PNA staining

Fresh testicular tissues were washed with ice-cold PBS and kept at − 80 °C until assay. Paraffin-embedded testis was sectioned at a thickness of 5 μm and stained with hematoxylin and eosin (H&E). Johnsen’s score is a well-established method for evaluating spermatogenic function [18] and the severity of germ cell injury was quantified by Johnsen’s score. Briefly, each section was given a score from 1 to 10 according to the following criteria: Score 10: Complete spermatogenesis with many spermatozoa. Germinal epithelium organized in a regular thickness leaving an open lumen. Score 9: Many spermatozoa present but germinal epithelium disorganized with marked sloughing or obliteration of the lumen. Score 8: Only few spermatozoa (< 5–10) present in the section. Score 7: No spermatozoa but many spermatids present. Score 6: No spermatozoa and only few spermatids (< 5–10) present. Score 5: No spermatozoa, no spermatids but several or many spermatocytes present. Score 4: Only few spermatocytes (< 5) and no spermatids or spermatozoa present. Score 3: Spermatogonia are the only germ cells present. Score 2: No germ cells but Sertoli cells are present. Score 1: No cells in the tubular section. The mean point value was from at least 10 seminiferous tubules.

Since PNA selectively binds to acrosome of sperms and can be used as a marker of sperm or haploid spermatid and further to evaluate integrity and numbers of sperms [19, 20]. PNA staining was conducted according to the manufacturer’s instructions. Generally, frozen slices were incubated with PNA (1:200) which conjugated with Alexa Fluor™ 594 (Life Technology) at room temperature for 30 min, washed 3 times with PBS. Nuclei were stained with DAPI (Sigma-Aldrich) for 5 min. Inverted fluorescence microscopy or confocal laser scanning microscopy was used to capture the image.

### Immunofluorescence staining

For immunofluorescence analyses, ice frozen slices were blocked with 10% donkey serum (Jackson, 017-000-121) for 0.5 h and then incubated with the primary antibodies: rabbit anti-MPO (Abcam, ab9535), rabbit anti-CD62E (Selectin-E, Abcam, ab18981), rabbit anti-Ki67 (Abcam, ab15580), rabbit anti-DDX4 (Abcam, ab13840) at 4 °C overnight. After washing with PBS three times, secondary antibodies, donkey anti-rabbit conjugated with Alexa 488(1:200, Life Technology), were incubated for 1 h at room temperature and then washed with PBS three times, nuclei were stained with DAPI. Inverted fluorescence microscopy was used to capture the image. The quantification results were evaluated in at least six representative visual fields for each group in a blinded manner by an experienced pathologist. Image-Pro Plus 6.0 (Media cybernetics, Silver Springs, MD, USA) was employed for image analysis.

### Assessment the levels of ROS in testis

ROS is the indicator of oxidative stress level and can be used to evaluate the oxidative environment of testicular tissues. In situ visualization of ROS production was assessed by 2′,7′-dichlorodihydro fluorescein diacetate (DCFH-DA,10 μmol/L, Invitrogen, C6827) histochemistry. Nuclei were stained with DAPI before captured the image under microscopy. Fluorescence intensity of staining was measured by ImageJ. Six representative visual fields of each group were counted.

### The effect of hUC-MSC over the HUVECs in vitro

We firstly collected the CM of hUC-MSC as follows: cells were cultured in complete medium up to 90% confluency, the cells were washed with PBS and changed with α-MEM basic medium (Life Technology) for another 24 h, and the medium was collected as hUC-MSC-CM. Then HUVECs were cultured to 90% confluency in ECM complete medium containing ECM medium (Life Technology) with 10% FBS and then changed with mixed medium which contained 50% ECM complete medium and 50% hUC-MSC-CM. ECM medium supplemented with 5% FBS was used as a control medium. Ten nanograms/milliliter TNF-α was used to stimulate HUVECs for 24 h. The HUVECs and cell supernatant were then collected to assess the expression of TNF-α, IL-1β, p-P65, and p-P38 and Selectin-E by real-time PCR and ELISA.

### RNA isolation and real-time quantitative PCR

For RNA isolation, cells or testicular tissues were harvested and total RNA of samples was extracted using the TRIzol reagent and reverse transcribed into cDNA using the PrimeScript RT Reagent Kit (TAKARA, RR037A) according to the manufacturer’s protocols. Real-time PCR was conducted with ChamQ Universal SYBR qPCR Master Mix (Vazyme, Q711-02) using Lightcycler 480 II (Roche) and normalized by the expression level of β-Actin. The information of primers was as follows:

β-Actin (human): forward sequence (5′-3′) GGACATCCGCAAAGACCTGTA, reverse sequence (5′-3′) GCATCCTGTCGGCAATGC. TNF- α (human): forward sequence (5′-3′) CCTCTCTCTAATCAGCCCTCTG, reverse sequence (5′-3′) GAGGACCTGGGAGTAGATGAG. IL-1β (human) forward sequence (5′-3′) ATGATGGCTTATTACAGTGGCAA, reverse sequence (5′-3′) GTCGGAGATTCGTAGCTGGA. CD62E (Selectin-E, human) forward sequence (5′-3′), reverse sequence (5′-3′) CCTTTGCTGACAATAAGCACTGG. β-Actin (rat): forward sequence (5′-3′) TGTCACCAACTGGGACGATA, reverse sequence (5′-3′) GGGGTGTTGAAGGTCTCAAA. TNF- α(Rat):forward sequence (5′-3′) CGCCACGAGCAGGAATGAGAAG, reverse sequence (5′-3′) GCATGATCCGAGATGTGGAACTGG. IL-1β(Rat): forward sequence (5′-3′) CACACTAGCAGGTCGTCATCATCC, reverse sequence (5′-3′): ATCTCACAGCAGCATCTCGACAAG. CD62E (Selectin-E, Rat): forward sequence (5′-3′): GGTCTGCGATGCTGCCTACTTG, reverse sequence (5′-3′): GAAGTGAGGTTGCTGCCACAGAG.

### Western blot

Testicular tissues or HUVECs were lysed in RIPA (Thermo) with phosphatase inhibitor (Merck) and protease inhibitor (Merck) for 30 min. Total protein concentration was measured by BCA protein assay kit (Thermo Fisher). The PVDF membranes transferred by proteins were blocked in 5% nonfat powder milk and then incubated with primary antibodies against Selectin-E, P65, P38, β-Actin, Caspase 3, and β-tubulin (all from Cell Signaling Technology) overnight at 4 °C. After washing with PBS three times, secondary antibody (Cell Signaling Technology) was incubated for 1 h at room temperature.

### ELISA test

IL-1β concentration in the supernatant of HUVECs was detected by IL-1β ELISA kit (R&D) according to the manufacturer’s instructions.

### Protein chip detection of secretions

The secretory protein in CM of hUC-MSC was accessed by human Antibody Array 507 protein chip (Raybiotech, AAH-BLG-507, glass slide). The procedure was done according to the manual of manufacture. Fluorescence signals were scanned with a GenePix 4000B (Axon Instruments, GenePix version 5.0). For each array, protein intensity values were background subtracted, scaled by the internal control, and floored at 1 unit. Human foreskin fibroblast cells (HEF) derived CM was used as control. HEF were isolated from Human discarded foreskin tissues and cultured as previously described [21]. The foreskin tissues were obtained from the circumcision upon the approval from the Institutional Ethical Review Committee of Shanghai Children’s Medical Center, Shanghai Jiao Tong University School of Medicine. Written informed consent was obtained from the patient and guardian.

### Statistical analysis

Data were presented as mean ± SEM at least 3 experiments. The statistical analysis was conducted by *t* test. *P* value lower than 0.05 was considered significant. Statistical analysis was assessed by SPSS software 22.0. Quantification of fluorescence intensity was accessed by ImageJ.

## Results

### hUC-MSC protect testes against I/R injury

**Fig. 1:**
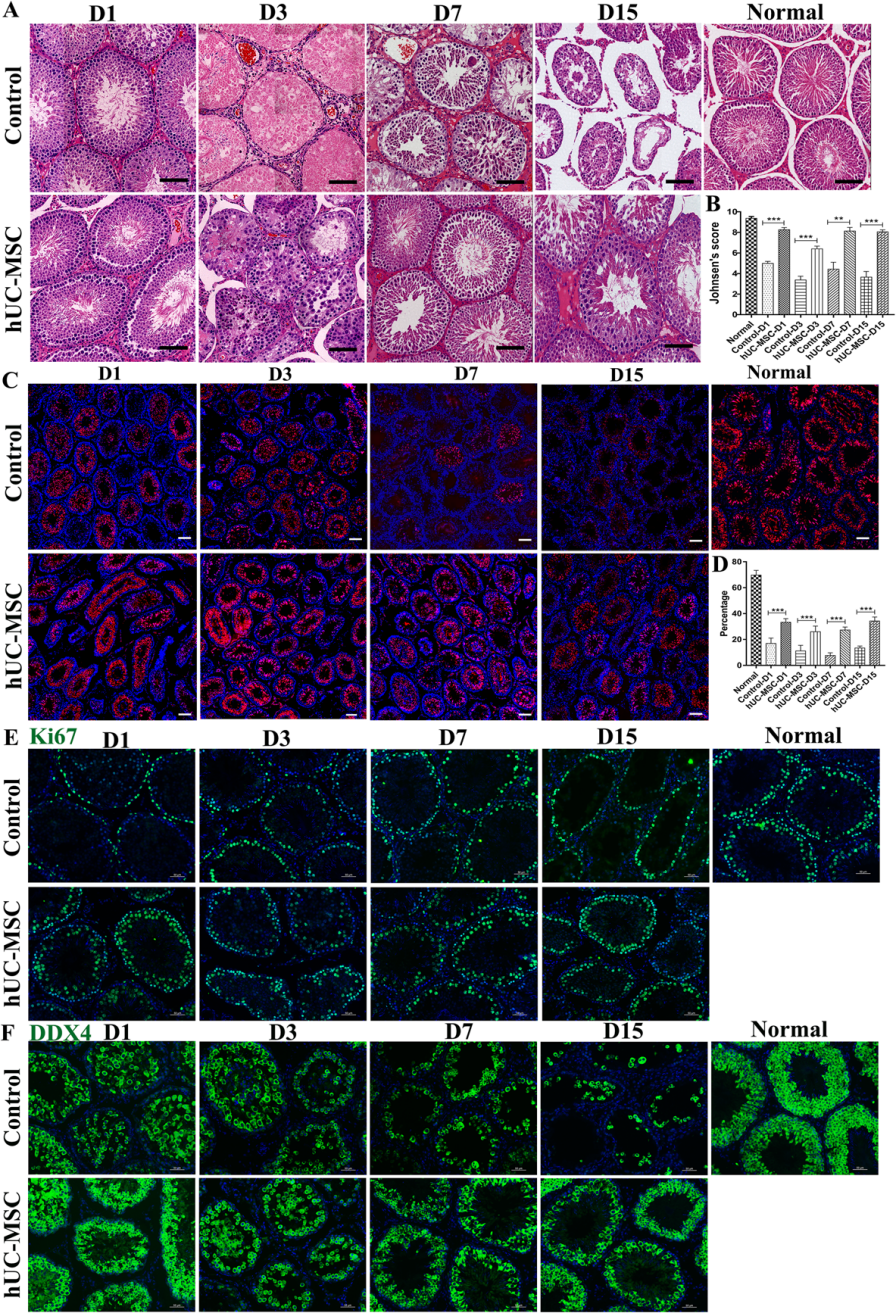
hUC-MSC alleviated spermatogenic cells injury during testicular torsion and detorsion. **a** H&E staining of rat testicular tissues at day 1 (D1), day 3 (D3), day 7 (D7), and day 15 (D15) after detorsion. The testes performed torsion and detorsion without hUC-MSC grafts were used as control. The normal group was untreated animals. Scale bars, 100 μm. **b** Johnsen’s score was evaluated at indicated day after hUC-MSC treatment. **c** Staining with PNA. Scale bars, 200 μm. **d** Quantification of seminiferous tubules containing PNA-positive cells. Ten representative sections of the pattern of testes were counted. At least three rats were used in every group. Data were represented as mean ± SEM. *P < 0.05, **P < 0.01, ***P < 0.001. **e** Immunostaining of rat seminiferous tubules at indicated day after torsion with proliferation marker Ki67. Scale bars, 50 μm. **f** Immunostaining of rat seminiferous tubules at indicated day after torsion with germ cell marker DDX4. Scale bars, 50 μm

The histopathological images show that torsion-detorsion significantly damaged spermatogenic cells and reduced the Johnsen’s score, especially at day 3 after detorsion (Fig. 1a, b; Fig. S2). But the MSC-treated testes had a marked improvement in Johnsen’s score compared with that of control, suggesting that the hUC-MSC restore recipient spermatogenesis.

To further determine if hUC-MSC could protect recipient spermatogenesis, we analyzed recipient spermatogenesis by staining spermatozoa with lectin PNA conjugated with Alexa Fluor™ 594, which selectively binds to acrosome of sperms and can be used as a sperm or haploid spermatid marker [20]. Figure 1c, d shows that seminiferous tubules of PNA-positive cells in the testes of hUC-MSC-treated rats were significantly increased compared to those of the control group. The data suggested that MSC has a protective effect on the survival of sperms.

As spermatogonia cells are crucial competent for the initiation of spermatogenesis, we next decided to determine whether hUC-MSC affected spermatogonia cell biology including testicular stem cells. We performed immunostaining of the testis tissues with Ki67 antibody (a marker reflecting the proliferating of spermatogonia) and DDX4 (a marker of germ cells including undifferentiated and differentiated spermatogonia). As shown in Fig. 1e, f, more Ki67- and DDX4-positive cells were present in the testes of the hUC-MSC group than those in the control testes. Moreover, DDX4-positive cells were evidently much more in the testes of the hUC-MSC group at day 15 after transplantation. These data indicated that the hUC-MSC contributed to the recipient testicular stem cell niche and promoted the survival, proliferation, and differentiation of recipient spermatogonia cells.

### hUC-MSC reduce inflammatory response of spermatogenic cells induced by I/R

**Fig. 2:**
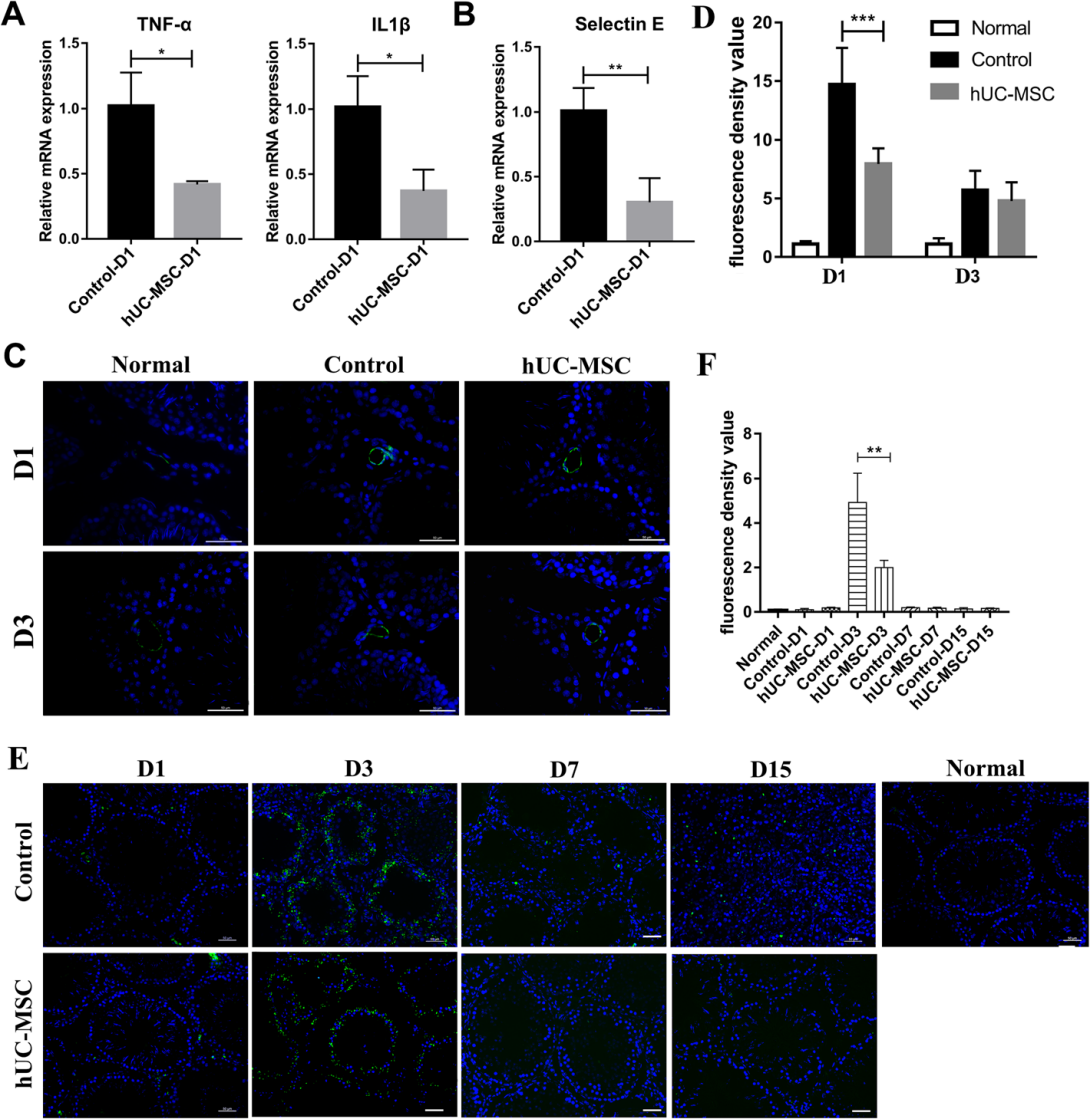
hUC-MSC injection reduced inflammatory factor production, Selectin-E expression, and neutrophil infiltration of testicular tissues in rats subjected I/R injury. **a** Real-time PCR analysis of mRNA expression of TNF-α and IL-1β in testicular tissues. The level of mRNA in control testes without hUC-MSC grafts was set as 1. Data was collected from at least 3 rats. **b** Relative mRNA expression of Selectin-E in testicular tissues. **c** Immunofluorescence staining of testicular tissues of rats with Selectin-E antibody. Scale bars, 50 μm. **d** Quantification of fluorescence density of Selectin-E in every seminiferous tubule. At least 10 representative sections of testes were counted. At least three rats were used in every group. Data were represented as mean ± SEM. E. Immunofluorescence staining of testicular tissues with MPO antibody. Scale bars, 50 μm. **f** Quantification of fluorescence density of MPO of every seminiferous tubule. At least 10 representative sections of testes were counted

Previous study reported that inflammatory response is the most important pathological mechanism during I/R injury of testicular detorsion [5]. Therefore, we first tested the mRNA levels of inflammatory factors TNF-α and IL-1β of testicular tissues by real-time PCR. hUC-MSC led to an obvious downregulation of mRNA levels of TNF-α and IL-1β at day 1 after detorsion compared to the control group (Fig. 2a). The data indicated that hUC-MSC reduced the expression levels of inflammatory factors of testis injured by I/R at day 1 after detorsion. In addition, Selectin-E is an endothelial cell adhesion molecule which mediates the adhesion of neutrophils [22]; thus, we detected the level of Selectin-E of testicular tissues by real-time PCR and immunofluorescence. As shown in the Fig. 2b, the mRNA level of Selectin-E of testicular tissues in the group of hUC-MSC was significantly lower than that of control at day 1 after detorsion. The immunostaining analysis obtained a similar result on the expression of Selectin-E at day 1 after detorsion (Fig. 2c, d). However, the expression of Selectin-E is not upregulated 3 days after detorsion.

Next, we further measured the neutrophil infiltration. Because testis is an immune cell-free organ, neutrophil infiltration may be the first response during the process of testicular I/R injury during torsion and detorsion operation; we tested the levels of MPO which was produced by neutrophils. As shown in Fig. 2e, f, the expression level of MPO was low at day 1 after detorsion, but high at day 3 after detorsion, and again returned to low level at day 7 and day 15 after detorsion, indicating that neutrophil infiltration happened at least 1 day after detorsion and reached its peak approximately at day 3. Furthermore, the level of MPO of testes in the hUC-MSC group was significantly lower than that of control. The results suggested that hUC-MSC remarkably reduced the levels of inflammatory factors and neutrophil infiltration of testes, resulting in a significant reduction of inflammatory response.

### hUC-MSC reduce the acute oxidative stress of spermatogenic cells induced by I/R injury

**Fig. 3:**
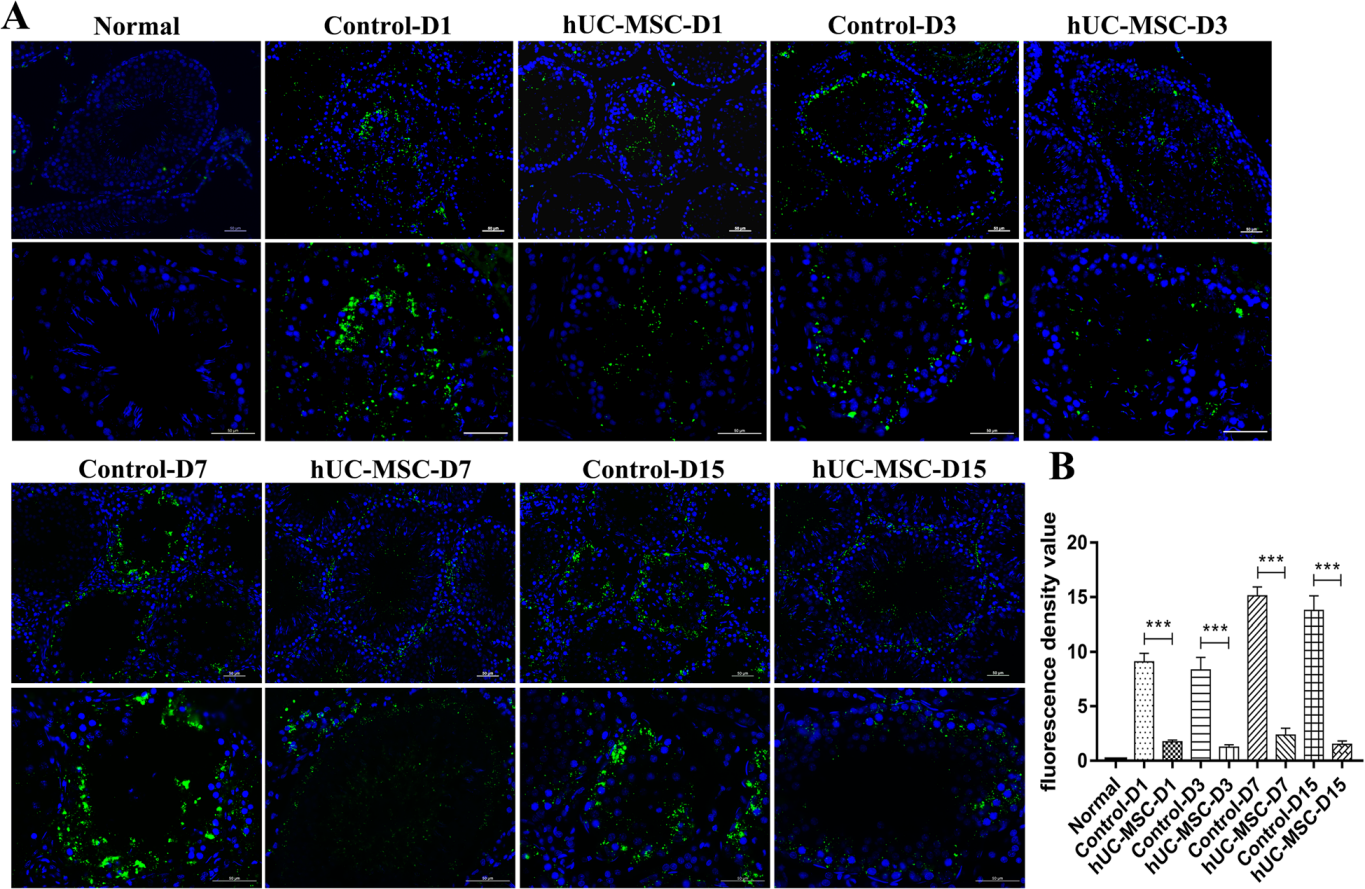
hUC-MSC reduced ROS production. **a** Immunofluorescence staining of testicular tissues of rats with ROS probe DCFH-DA. Scale bars, 50 μm. **b** Quantification of fluorescence intensity of ROS in every seminiferous tubule. At least 10 representative sections of the testes were counted. At least three rats were used in every group. Data were represented as mean ± SEM

Previous research showed that ROS during I/R could cause DNA damage, endothelial damage, and germinal cell apoptosis [7]. In this study, we wonder if hUC-MSC could reduce the acute oxidative injury. Figure 3 shows that ROS were over-produced during I/R injury of testes, but the ROS level of testicular tissues in hUC-MSC-treated group was evidently lower than that of control at day 1 to day 15 after detorsion. The above results indicated that hUC-MSC significantly reduced ROS levels during I/R injury of spermatogenic cells.

### hUC-MSC protect germ cells against apoptosis induced by I/R injury

**Fig. 4:**
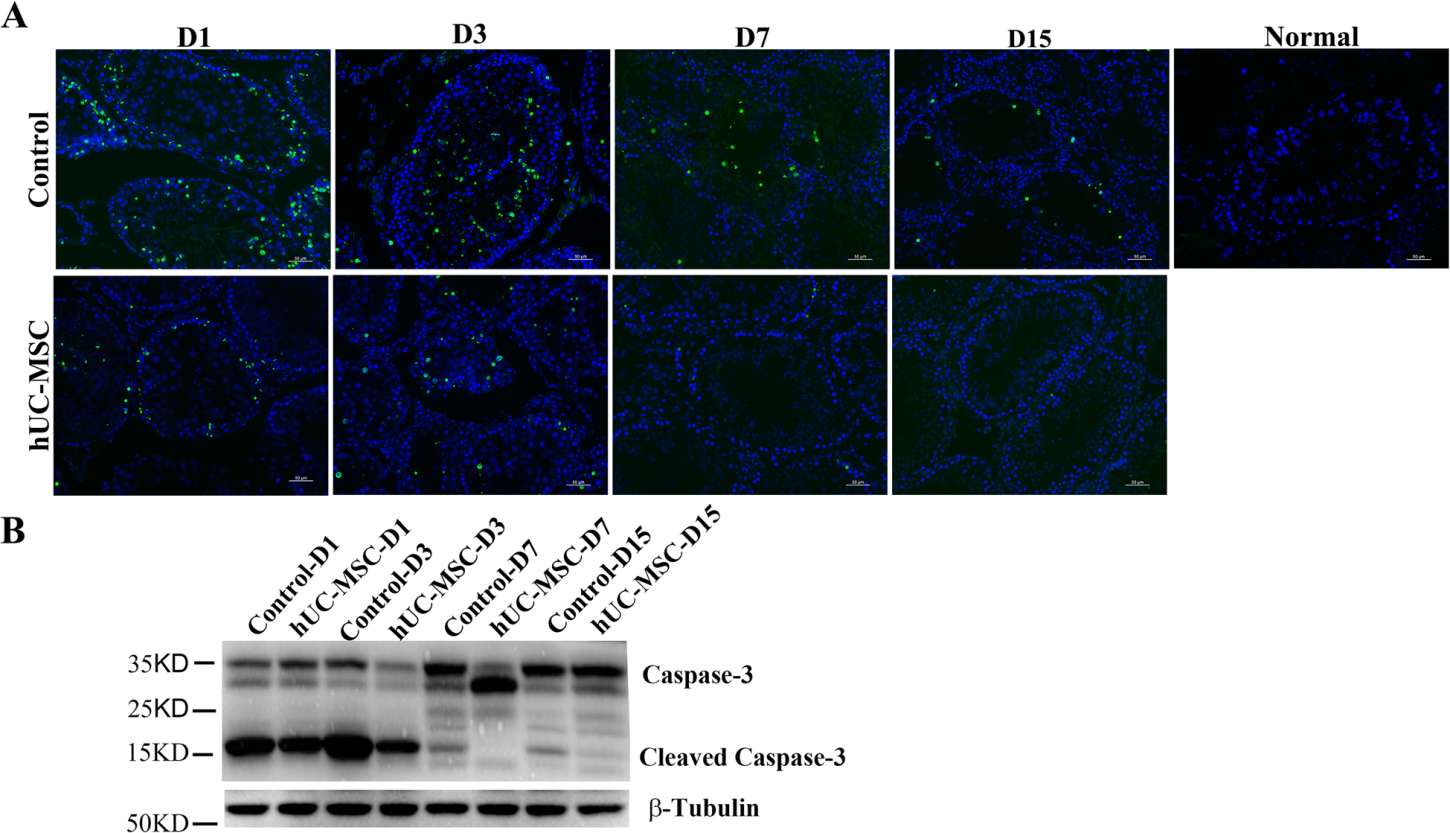
hUC-MSC protected germ cells against I/R induced apoptosis. **a** TUNEL assay of testicular tissues after day 1 to day 15 after torsion. Scale bars, 50 μm. **b** Western blot analysis of testicular tissues at indicated days with antibodies against caspase 3 and β-tubulin. The protein level of β-tubulin was used as internal

Testicular torsion can lead to germ cell apoptosis and apoptotic pathways are often activated by inflammation and ROS [6, 23]. Next, to determine whether hUC-MSC could protect germ cells against torsion-induced apoptosis and restore spermatogenesis, TUNEL assay and western blot were performed. Immunofluorescence staining with apoptosis marker TUNEL showed that testicular torsion induced germ cell apoptosis especially at day 1 and day 3 after detorsion, while testes in the normal group were negative for TUNEL (Fig. 4a). However, a significant reduction of germ cell apoptosis appeared in the group of hUC-MSC than the control group at day 1 and day 3 after detorsion (Fig. 4a). Of note, the level of TUNEL was low 7 days later after detorsion in both the control and hUC-MSC group. And more, germ cells in the seminiferous tubes were fewer in the control group than those of the hUC-MSC group (Figs. 4a and 1f). The data revealed that the apoptotic germ cells were dead 7 days after detorsion. Additionally, western blot analysis showed that the level of cleaved caspase 3 was lower than that of the control group (Fig. 4b). Taken together, hUC-MSC reduced germ cell apoptosis induced by I/R injury and promote spermatogenesis.

### The CM of hUC-MSC reduce inflammatory response of HUVECs in vitro

**Fig. 5:**
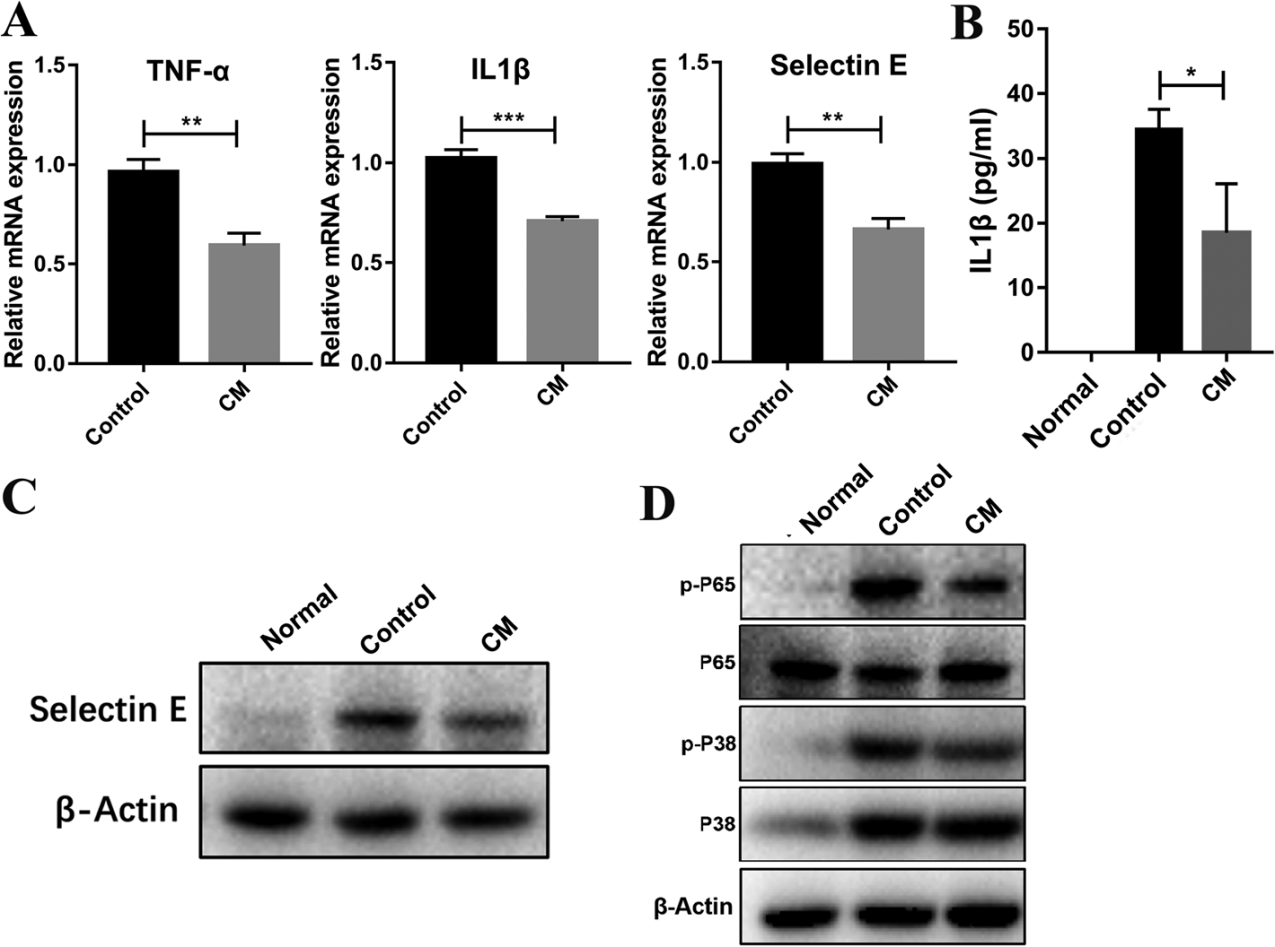
hUC-MSC-CM inhibits inflammatory factors production and inflammatory pathway in HUVECs. HUVECs stimulated by 10 ng/ml TNF-α were cultured with or without hUC-MSC-CM. Twenty-four hours later, the cells were harvested for RNA extract. **a** Real-time PCR analysis of mRNA levels of TNF-α, IL-1β, and Selectin-E of HUVECs. The level of mRNA in HUVECs was set as 1. Data was collected from at least 3 separated experiments. **b** ELISA analysis of IL-1β concentration in the supernatant of HUVECs. **c** Western blot analysis of Selectin-E expression of HUVECs with Selectin-E and β-actin antibodies. **d** Western blot analysis of P65 and P38 activation of HUVECs with antibodies against P-P65, P65, P-P38, P38, and β-actin. CM represents hUC-MSC-CM

Previous studies demonstrated that MSC could produce many paracrine factors contributing to the protection of cells against injury [24, 25]. Thus, we wondered if the CM of hUC-MSC (hUC-MSC-CM) could reduce inflammatory response of HUVECs induced by TNF-α. As shown in Fig. 5a, hUC-MSC-CM can reduce the mRNA levels of TNF-α, IL-1β, and Selectin-E of HUVECs stimulated by TNF-α. And more, the concentration of IL-1β protein in the supernatant of HUVECs was also decreased in the hUC-MSC group (Fig. 5b). In addition, the protein level of Selectin-E was also decreased in the hUC-MSC-CM-treated group (Fig. 5c). Next, we would further determine if the inflammatory pathway was activated when HUVECs stimulated by TNF-α. Western blot results showed that the expression of p-P65 and p-P38 was evidently upregulated when HUVECs were stimulated by TNF-α. However, hUC-MSC-CM could downregulate the level of p-P65 and p-P38 (Fig. 5d). These data suggested that hUC-MSC could reduce inflammatory response by their paracrine factors in vitro.

### Analysis of paracrine factors of hUC-MSC

**Fig. 6:**
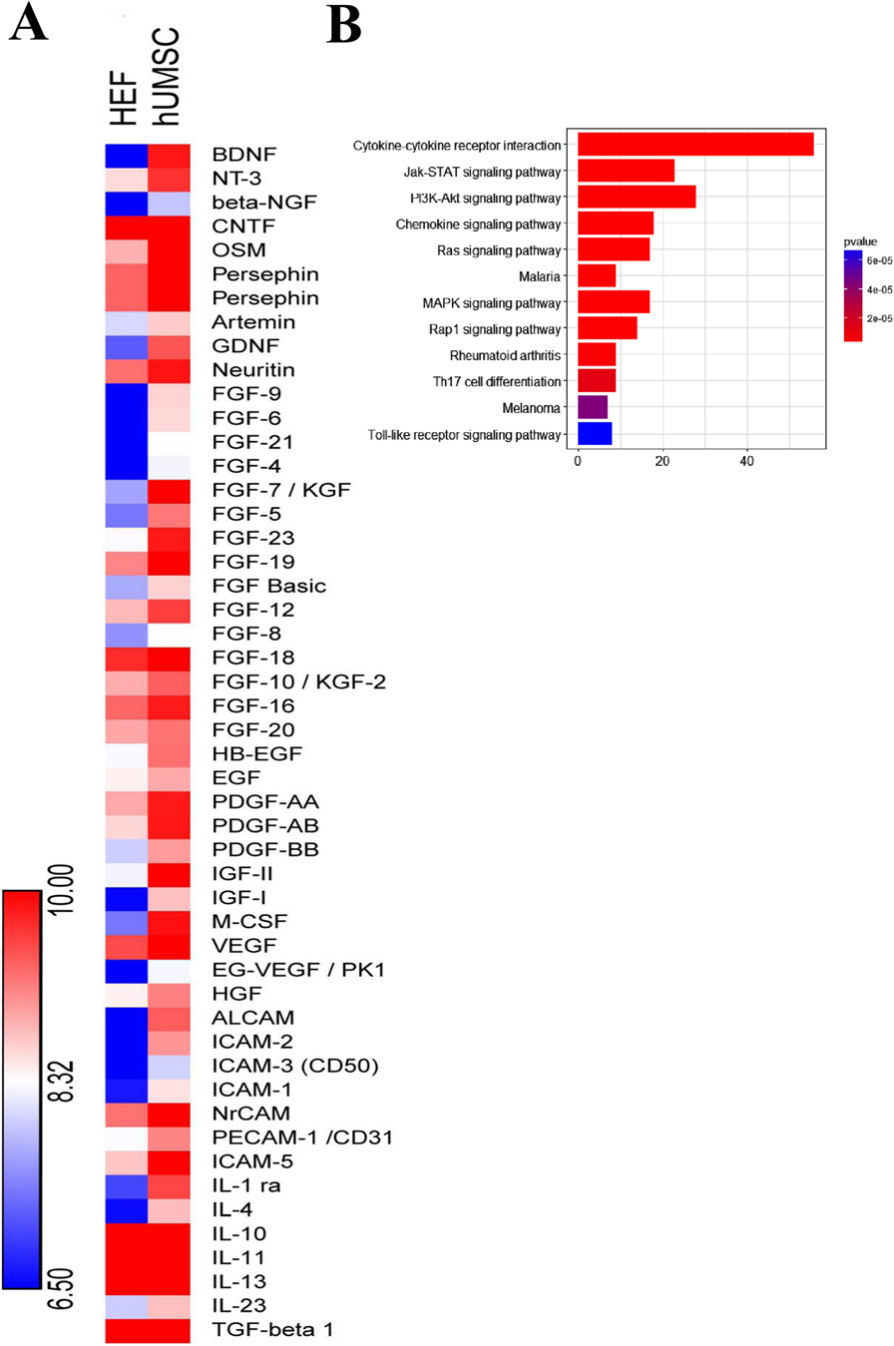
Normalized array data of the 507 proteins were analyzed by SAM to detect a difference in their concentrations between the hUC-MSC-CM and HEF-CM. **a** The relative concentrations of the neurotrophic factors, growth factors, cell adhesion molecules, and anti-inflammatory factors that obtained a significant score (*q* value < 0.001%) are shown in a “heatmap.” Low concentrations are shown in blue, medium concentrations in white and high concentrations in red. Also, see Table S1. **b** KEGG pathway analysis of the soluble factors in the CM of hUC-MSC and hEF. Enriched pathways in the CM of hUC-MSCs that obtained a significant score (*P* value < 0.05). HEF represents hEF-CM. hUMSC presents hUC-MSC-CM

To determine what kinds of paracrine factors of hUC-MSC-CM are beneficial to lesioned spermatogenic cells, we analyzed paracrine factors in both hUC-MSC-CM and HEF-CM (as a control) by the human antibody array kit against 507 soluble proteins. The data showed that the hUC-MSC-CM and HEF-CMs differed in the protein levels (Table S1). Anti-inflammatory cytokines IL1ra, IL10, IL13, TGF-β1, and nutritive cytokines BDNF, GDNF, CNTF, HGF, FGF, EGF, and VEGF were richer in the hUC-MSC-CM compared to HEF-CM (Fig. 6a). Interestingly, HGF, which is well known to play a regulatory role of Selectin-E, was also richer in the hUC-MSC-CM. Furthermore, KEGG pathway enrichment analysis revealed that several pathways including JAK-STAT, PI3K-Akt signaling, and MAPK signaling pathway are activated in the hUC-MSC-CM (Fig. 6b), which are crucial signaling pathways to germ cell proliferation and differentiation. Altogether, the results suggested that the anti-inflammatory factors and other growth factors could contribute to the protective effects of hUC-MSC against I/R injury of spermatogenic cells.

## Discussion

Testicular torsion involving rotation of the testis and twisting of the spermatic cord will cause testicular atrophy. An immediate detorsion operation is required to prevent testicular ischemic necrosis within 4 to 8 h after torsion [26]. I/R injury during testicular torsion and detorsion operation of rat testis could result in a permanent loss of spermatogenesis despite the return of blood flow. Recently, MSCs are reported to be effective in attenuating myocardial I/R injury in rats [27]. In this study, we found that hUC-MSC injected intravenously into rats subjected to testicular torsion and detorsion operation could attenuate I/R injury and promote the proliferation and differentiation of spermatogonia cells resulting in the survival and the regeneration of more spermatogenic cells and sperms (Fig. 1).

Many studies have demonstrated that MSCs have immunosuppressive activities owing to their paracrine effects and interactions with immune cells, which will subsequently reduce the level of TNF-α and IL-1β [28, 29]. Subsequently, TNF- α and IL-1 β can activate JNK signaling pathway which leads to the expression of Selectin-E in endothelial cells and ultimately results in the recruitment of neutrophil [22, 30, 31]. In our study, the expression level of inflammatory factors TNF-α and IL-1β was upregulated during I/R of the testis at day 1 after torsion, and more, hUC-MSC significantly decreased the expression level of inflammatory factors TNF-α and IL-1β at day 1 after torsion (Fig. 2a). However, the expression level of TNF-α and IL-1β did not increase compared to those of the normal group 3 days after detorsion (data not shown). Moreover, Selectin-E was upregulated during I/R of the testis and hUC-MSC evidently reduced the expression level of Selectin-E at day 1 after torsion. Subsequently, the neutrophil infiltration began to appear mainly at day 3 after detorsion and then returned to a low level, and hUC-MSC significantly reduced the neutrophil infiltration at day3 after detorsion (Fig. 2). Subsequently, the expression level of Selectin-E returned to a low level 3 days after testis detorsion (Fig. 2b–d). Consistent with our study, the downregulation of TNF- α and IL1 β lead to the decline of Selectin-E gene expression [32–35]. In addition, neutrophil infiltration mainly appeared at day 3 after detorsion and then began to fall at day 7 and day 15 after detorsion evidenced by the expression of MPO was highest at day 3 after testis detorsion and became low again at day 7 and day 15(Fig. 2e, f). The data indicated that inflammatory response during the testis I/R injury is an early pathological phenomenon and hUC-MSC could reduce the inflammatory response and promote the survival and regeneration of germ cells. In addition, in vitro hUC-MSC-CM had a similar inflammatory suppressive effect on HUVECs stimulated by TNF-α, evidenced by decreased levels of TNF-α, IL-1β, and Selectin-E of HUVECs and P65, P38 phosphorylation (Fig. 5). This result indicated that hUC-MSC can reduce inflammatory response by paracrine factors.

As for molecular mechanism, many anti-inflammatory cytokines, such as IL-1ra, IL10, IL13, and TGF-β1, were detected by protein chip and rich in the CM of hUC-MSC (Table S1 and Fig. 6). Of note, IL-1ra, IL10, and IL13 are demonstrated to reduce inflammatory response [36–39]. Therefore, the anti-inflammatory cytokines may contribute to the immunosuppressive function of hUC-MSC on I/R injury of spermatogenic cells. In addition, the HGF in the CM of hUC-MSC may play a role on the inhibition of the expression of Selectin-E. Previous studies revealed that HGF can inhibit neutrophil infiltration via the downregulation of Selectin-E on the endothelial cell surface, which suppresses ischemia-related injury in various organs [40–42]. Thus, HGF secreted by hUC-MSC may downregulate Selectin-E to reduce neutrophil infiltration. In summary, hUC-MSC could reduce inflammatory response by secreting anti-inflammatory factors, HGF, and other factors.

Additionally, ROS is produced through normal metabolic reactions and have roles in the processes such as pathogen killing and cell signaling [43]. Overgeneration of ROS has been associated with I/R injury in different organs and testes are highly sensitive to ROS damage [44]. In this study, ROS was over-produced during I/R injury of testes at day 1 to day 15 after torsion (Fig. 3). But hUC-MSC could significantly reduce the level of ROS, indicating that hUC-MSC could decrease oxidative stress and oxidative injury during testis torsion and detorsion.

On the other hand, previous studies found that testis torsion could induce germ cell apoptosis. In the testis torsion and detorsion animal experiment, germ cell apoptosis was evident at day 1 and day 3 after torsion; however, it was reduced at day 7 and day 15 after torsion. The data revealed that testis torsion first induced germ cell apoptosis; subsequently, the apoptotic germ cells were dead, evidenced by fewer germ cells (DDX4-positive cells) in the seminiferous tubules in the control group (Figs. 1 and 4a). Interestingly, hUC-MSC protected germ cells against torsion-induced apoptosis and restored spermatogenesis (Figs. 1 and 4a).

Stem cells can be transplanted through local injection or intravenous injection. Though local injection can bypass the blood-testis barrier and directly affect the testis, it is not a good treatment option in clinical practice. The potential risk of compartment syndrome by local injection has limited its clinical application [11, 12]. In our previous study of I/R injury of the kidney, intravenous stem cells still conducted a great protective effect though they were blocked in the lung [13]. In the present study, hUC-MSC possibly played a suppressive role on inflammatory response and decline of acute oxidative injury via the paracrine factors. Interestingly, we found a large number of nutritive cytokines and anti-inflammatory factors in the CM of hUC-MSC. They may be able to enter the testis via the circulation and reduce inflammatory response and ROS and support the survival and growth of testicular cells during I/R injury of testes.

## Conclusions

In summary, this study demonstrated that hUC-MSC could protect the spermatogenic cells against I/R injury. And more, hUC-MSC could reduce inflammatory response evidenced by downregulating the expression of inflammatory factor and infiltration of neutrophils. Furthermore, hUC-MSC ameliorated acute oxidative injury by reducing the ROS level. Besides, hUC-MSC protected germ cell apoptosis induced by I/R injury and promoted spermatogenesis. Paracrine factors secreted by MSC may be a main mechanism. Therefore, the present study provides a method for clinical treatment of I/R injury during testes detorsion operation to facilitate the spermatogenesis.

## Supplementary information

**Additional file 1: Figure S1.** Presentation of testicular I/R rat model method. The testicular I/R rat model was established through 720-degree torsion for 1 h and the testes became purple, then the testes returned to red from purple or black after detorsion. hUC-MSC were intravenously injected 10 min before detorsion. Only the testes which return to red after detorsion will be used in the further study.

**Additional file 2: Figure S2.** hUC-MSC alleviated spermatogenic cells injury during testicular torsion and detorsion. (A, B) The images of the entire section of mouse testes at indicted time after torsion by H&E staining(A) and PNA staining (B). Scale bars:2000 μm.

**Additional file 3: Table S1.** A table of normalized fluorescence signal intensity of neurotrophic factors, growth factors, cell adhesion molecules (CAM) and anti-inflammatory factors in the HEF-CM or hUC-MSC-CM and ratio of them.

## Data Availability

All data generated or analyzed during this study are included in this published article and its supplementary information files.

## Abbreviations

hUC-MSC: Human umbilical cord mesenchymal stromal cells
I/R: Ischemia/reperfusion
ROS: Reactive oxidative species
DCFH-DA: 2′,7′-Dichlorodihydro fluorescein diacetate
CM: Condition medium
HUVECs: Human umbilical vein endothelial cells
MEMα: Minimum essential medium α
FBS: Fetal bovine serum
H&E: Hematoxylin and eosin
MPO: Myeloperoxidase
HEF: Human foreskin fibroblast cells
