# Supplementary material for: Human umbilical cord multipotent mesenchymal stromal cells alleviate acute ischemia-reperfusion injury of spermatogenic cells via reducing inflammatory response and oxidative stress
Source: Stem Cell Res Ther. 2020 Jul 17;11:294. doi: 10.1186/s13287-020-01813-5 (PMC7366899; doi:10.1186/s13287-020-01813-5)
Supplement: Supplementary file 3 — Additional file 3: Table S1. A table of normalized fluorescence signal intensity of neurotrophic factors, growth factors, cell adhesion molecules (CAM) and anti-inflammatory factors in the HEF-CM or hUC-MSC-CM and ratio of them. [file 13287_2020_1813_MOESM3_ESM.docx]

Supplemental Information

Figure S1. Presentation of testicular I/R rat model method. The testicular I/R rat model was established through 720-degree torsion for 1 hour and the testes became purple, then the testes returned to red from purple or black after detorsion. hUC-MSC were intravenously injected 10 minutes before detorsion. Only the testes which return to red after detorsion will be used in the further study.

Figure S2. hUC-MSC alleviated spermatogenic cells injury during testicular torsion and detorsion. (A, B) The images of the entire section of mouse testes at indicted time after torsion by H&E staining(A) and PNA staining (B). Scale bars:2000μm.

**Table S1:** A table of normalized fluorescence signal intensity of neurotrophic factors, growth factors, cell adhesion molecules (CAM) and anti-inflammatory factors in the HEF-CM or hUC-MSC-CM and ratio of them.
